# Fabricating Zein-OSA Starch Complexes as Multifunctional Carriers for Carrot Oil

**DOI:** 10.3390/foods15030435

**Published:** 2026-01-24

**Authors:** Lei Chen, Bin Li, Zhanhang Ye, Yexin Shen, Hui Teng, Yanan Zhao

**Affiliations:** 1 College of Food Science and Technology, Guangdong Ocean University, Zhanjiang 524000, China; 2 Guangdong Provincial Key Laboratory of Aquatic Product Processing and Safety, Zhanjiang 524000, China; 3 Guangdong Province Engineering Laboratory for Marine Biological Products, Zhanjiang 524000, China; 4 Guangdong Provincial Engineering Technology Research Center of Seafood, Zhanjiang 524000, China; 5 Key Laboratory of Advanced Processing of Aquatic Product of Guangdong Higher Education Institution, Zhanjiang 524000, China; 6 Shenzhen Institute, Guangdong Ocean University, Shenzhen 518108, China

**Keywords:** emulsion, carrot oil, Zein, OSA starch

## Abstract

This study tackles the stabilization and delivery challenges of oxidation-prone carrot oil by engineering tailored Zein-OSA starch hybrid complexes. The influence of complex mass ratios (1:2, 1:1, 2:1) on key structural, colloidal, and functional properties was meticulously evaluated. The complexes were analyzed through spectroscopy, thermal methods, and microscopy. Derived emulsions were assessed for stability under environmental stresses (pH, salts, storage), alongside their rheological behavior and aroma retention. The 1:1 complex emerged with optimal molecular compatibility, thermal stability, and barrier properties. In emulsions, the 1:2 formulation provided the most uniform droplets and superior salt tolerance, while the 1:1 ratio yielded the best pH stability. All emulsions were shear-thinning. Microencapsulation effectively converted the emulsion into a stable, free-flowing powder. This work demonstrates a rational approach to designing robust plant-based delivery systems for protecting and improving the functionality of sensitive lipophilic ingredients in practical applications.

## 1. Introduction

Carrot (*Daucus carota* L.) seeds, often considered an agricultural by-product, represent an underutilized botanical resource of significant nutritional and functional value. Comprising approximately 17% lipids, 17% proteins, and 10% carbohydrates, they are a rich source of bioactive compounds [1]. The oil derived from these seeds is particularly noteworthy for its distinctive composition and associated health-promoting properties. It is characterized by a rich profile of aromatic and phenolic compounds, phytosterols, and a high proportion of long-chain unsaturated fatty acids (predominantly C16–C20), such as oleic, linoleic, and linolenic acids [2,3]. This unique composition confers antioxidant, anti-inflammatory, and potential cardioprotective activities, underpinning the growing interest in carrot oil as a versatile ingredient in the nutraceutical, cosmetic, and functional food industries [4]. Specifically, its potential extends to serving as a natural lipid phase for the stabilization and delivery of sensitive bioactive compounds. However, the translation of this potential into practical applications is severely constrained by several inherent physicochemical limitations. Like many plant-derived essential oils and lipophilic bioactives, carrot oil suffers from poor aqueous solubility, which limits its incorporation into water-based products [5]. Furthermore, it exhibits high volatility and pronounced susceptibility to degradation triggered by environmental factors such as heat, light, and oxygen [6]. These instabilities lead to rapid loss of volatile aromatic compounds, oxidative rancidity, and a consequent diminution of their bioactivity and commercial value during processing and storage. Therefore, developing effective encapsulation and delivery systems to enhance its stability, mask volatility, improve water dispersibility, and enable controlled release constitutes a primary technological challenge for unlocking its full commercial and functional potential.

To address these challenges, emulsion-based delivery systems have emerged as a predominant strategy. An emulsion is a kinetically stabilized mixture of two immiscible liquids, typically classified as oil–in–water (O/W) or water–in–oil (W/O) based on the nature of the continuous phase [7]. These systems can effectively encapsulate lipophilic compounds, improve their bioavailability, and provide a degree of protection from the environment. Beyond conventional emulsions stabilized by low-molecular-weight surfactants, particle-stabilized emulsions, known as Pickering emulsions, have gained considerable attention for their superior stability [8]. Pickering emulsions are stabilized by solid particles that adsorb irreversibly at the oil–water interface, forming a rigid physical barrier that prevents droplet coalescence. This often results in enhanced stability against Ostwald ripening, coalescence, and environmental stresses compared to surfactant-stabilized counterparts [9,10]. In this context, food-grade biopolymer particles, particularly protein–polysaccharide complexes, have become promising Pickering stabilizers due to their biocompatibility, biodegradability, and functional versatility. Zein, the major prolamin protein from corn, is highly hydrophobic and has excellent film-forming and adhesive properties, making it an effective material for binding and encapsulating lipophilic oils [11]. Notably, Zein also possesses inherent antioxidant activity, which can provide primary protection against lipid oxidation [12]. However, Zein particles alone often lead to aggregates with limited colloidal stability in aqueous systems. To overcome this, a combination with polysaccharides is a common strategy. Octenyl succinic anhydride (OSA)-modified starch, a widely used amphiphilic polysaccharide, acts as an outstanding emulsifying stabilizer. Its introduction introduces steric and electrostatic repulsion, significantly improving the dispersibility and long-term physical stability of composite particles in aqueous phases [13,14]. The synergistic combination of Zein and OSA starch can thus create composite particles with optimized interfacial activity, antioxidant capacity, and colloidal stability, representing a sophisticated platform for encapsulating sensitive oils.

While the general encapsulation of plant oils within emulsion-based systems has been extensively studied, research specifically targeting carrot oil remains relatively scarce. Previous studies have explored its stabilization using conventional surfactants [4] or single biopolymers like gum Arabic [15]. However, systematic investigations into the design, fabrication, and stabilization performance of structured binary complexes, specifically tailored Zein-OSA starch composites at defined mass ratios for carrot oil encapsulation, are notably lacking. The optimization of such composite systems is crucial, as the ratio between protein and polysaccharide directly dictates the interfacial architecture, particle characteristics, and ultimately, the emulsion stability and functional performance of the encapsulates. To address this, we propose stabilizing carrot oil with Zein-OSA starch complexes. This work involves characterizing the complexes, assessing the stability of the resulting emulsions, and evaluating the properties of derived microcapsules. The microencapsulation approach represents a significant advancement over emulsion technology, providing enhanced protection and controlled release, thereby overcoming the drawbacks of liquid emulsions and broadening the application scope of functional encapsulates. This work aims to provide fundamental insights into the structure–function relationship of protein–polysaccharide stabilized interfaces for a valuable but challenging oil, and to develop a viable microencapsulation platform with substantial scientific and practical importance for the functional food and nutraceutical industries.

## 2. Materials and Methods

### 2.1. Materials

Octenyl succinic anhydride modified starch (OSA-starch) and Zein were procured from Shanghai Yuanye Bio-Technology Co., Ltd. (Shanghai, China). Carrot oil was obtained from Hongxing Natural Medicinal Flavor Oil Factory (Ji’an city, Jiangxi, China). The oil was stored in its original sealed, light-proof container at room temperature (approximately 25 °C) prior to use, and all experiments were completed within one month of opening the container to ensure freshness. All aqueous solutions were prepared using ultrapure water.

### 2.2. Synthesis of Zein-OSA Conjugates

Zein-OSA starch composite particles were synthesized based on a reported method from Dai et al. [16] with modifications. Briefly, Zein powder (5 g) was dissolved in 75% (*v*/*v*) aqueous ethanol (100 mL) under continuous stirring (600 rpm, 1 h). The solution pH was adjusted to 3.5 using 0.1 M NaOH. Subsequently, 20 mL of this solution was injected into 100 mL of acidified ultrapure water (pH 3.5) at a rate of 10 mL/min under stirring (600 rpm, 1 h) to form Zein gel particles. Separately, OSA starch dispersion was prepared by dissolving OSA starch powder (10 g) in ultrapure water (240 mL) at 50 °C, followed by stirring (600 rpm, 1 h), pH adjustment to 3.5, and cooling to room temperature. Zein particles were prepared via an anti-solvent precipitation method to obtain a dispersion with a final concentration of 0.1 g/mL. Simultaneously, an aqueous solution of OSA-modified starch was prepared at a concentration of 0.04 g/mL. The two components were then blended at Zein-OSA starch dry-mass ratios of 1:1, 1:2, and 2:1, respectively. The mixtures were prefrozen at −20 °C for 48 h and lyophilized to obtain the composite particles.

### 2.3. Structural Characterization of Zein-OSA Starch

#### 2.3.1. Particle Size and Zeta

The hydrodynamic particle size was determined by dynamic light scattering (DLS) using a ZETASIZER NANO ZSE instrument (Malvern Instruments Ltd., Malvern, UK) at 25 °C with a scattering angle of 90° and a refractive index of 1.46.

#### 2.3.2. Fluorescent Intensity

Fluorescence spectra were recorded on a Shimadzu RF-5301 spectrophotometer (Shimadzu, Kyoto, Japan). Samples were diluted to 1 mg/mL with deionized water. The excitation wavelength was set at 280 nm, and emission spectra were collected from 300 to 500 nm with slit widths of 5 nm for both excitation and emission.

#### 2.3.3. Fourier-Transform Infrared (FTIR) Spectroscopy Analysis

FT-IR spectra of Zein, OSA starch, and their complexes were acquired using a Nicolet iS5 spectrometer (Bruker TENSOR 27, Bremen, Germany). Lyophilized samples were mixed with KBr and pressed into pellets. Spectra were recorded from 400 to 4000 cm^−1^ at a resolution of 4 cm^−1^, averaging 64 scans at 25 °C.

#### 2.3.4. Differential Scanning Calorimetry (DSC)

Thermal analysis was performed on a simultaneous thermal analyzer (STA 449F3, Jupiter, Hamburg, Germany) following a method adapted from Huang et al. [17]. Samples were sealed in aluminum crucibles and heated from 30 to 150 °C at a rate of 10 °C/min under a nitrogen flow of 20 mL/min. An empty crucible served as the reference.

#### 2.3.5. Determination of Moisture Content

A gravimetric oven-drying method was employed to determine the moisture content. Samples (5 g) were dried at 105 °C in a convection oven. Drying continued until the difference between two successive weighings (with 1 h intervals) was less than 0.5 mg, which was defined as constant weight. The moisture content was calculated from the weight loss and expressed as a percentage (*w*/*w*). Triplicate measurements were conducted.

#### 2.3.6. Scanning Electron Microscopy (SEM)

Sample morphology was examined using a Phenom ProX scanning electron microscope (Phenom-World, Eindhoven, The Netherlands) under high vacuum. Samples were mounted on adhesive tape, sputter-coated with gold, and imaged at an accelerating voltage of 10 kV.

### 2.4. Preparation of Emulsion-Loaded Carrot Oil

**Figure 1:**
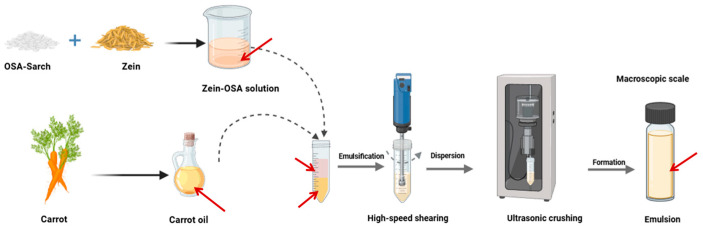
Emulsion preparation flow chart.

Carrot oil emulsions stabilized by the complexes were prepared based on a previous report, with modifications [18]. The oil phase (carrot oil) was mixed with an aqueous dispersion of the complexes at a volume ratio of 1:19, resulting in a final oil concentration of 5% (*v*/*v*) in the emulsion. The mixture was first homogenized at 10,000 rpm for 3 min at room temperature, followed by ultrasonication (300 W, 10 min) in an ice-water bath to form the final emulsion, as shown in Figure 1.

### 2.5. Characterization of Emulsion

#### 2.5.1. Microstructure Observation

Emulsion droplet size and morphology were observed using optical microscopy at 100× magnification. For confocal laser scanning microscopy (CLSM, Zeiss, Oberkochen, Germany), emulsions were dual-stained with Nile red (for oil) and Nile blue (for complexes). After incubation (5 min) and storage in the dark (2 h), samples were imaged using excitation wavelengths of 488 nm and 633 nm.

#### 2.5.2. Determination of Odor Release

The release of volatile compounds from emulsions stored under ambient conditions was monitored using an electronic nose (Win Muster Airsense Analysis, Schwerin, Germany), following the method described [19]. Each sample was analyzed in quintuplicate.

#### 2.5.3. GC-MS

Volatile flavor profiles of carrot oil and the emulsions were analyzed by headspace solid-phase microextraction coupled with GC-MS [20]. Separation was performed on a DB-WAX capillary column (30 m × 0.25 mm × 0.25 μm) with a helium flow rate of 1 mL/min. The mass spectrometer operated in electron impact (EI) mode at 70 eV. Compounds were identified by matching mass spectra against the NIST 17 library and the reported literature data.

#### 2.5.4. Changes in Emulsion During Storage

For storage stability, emulsions were kept at room temperature in the dark for 7 days. For pH stability, emulsions were adjusted to pH 2.0, 4.0, 6.0, 8.0, and 10.0 and observed after 2 h. For ionic stability, NaCl was added to achieve final concentrations of 0 to 0.5 M, and samples were observed after 2 h, under the same conditions as in our previous research [21]. Emulsion particle size was recorded throughout these stability tests.

#### 2.5.5. Rheological Properties of Emulsions

Flow behavior was analyzed using a HAAKE MARS modular rheometer (Thermo Fisher Scientific, Waltham, MA, USA) [17]. Approximately 1.0 mL of emulsion was subjected to a shear rate sweep from 0.1 to 100 s^−1^ at a constant strain of 1%, and the apparent viscosity was recorded.

### 2.6. Molecular Docking for Binding Interactions

Molecular docking was performed to study interactions between Zein and key components (OSA-starch, oleic acid, linoleic acid). The Zein structure was modeled using LOMETS (https://www.aideepmed.com/LOMETS/, accessed on 21 December 2025), and ligand structures were built using Chem3D(2023). All structures were processed (dehydration, hydrogenation) and converted to PDBQT format using AutoDockTools-1.5.7 for docking simulations [20].

### 2.7. Spray Drying of Emulsion and Characterization

The emulsion was mixed with maltodextrin (30% of total solids) to improve drying yield. Spray drying was conducted under the following specific parameters: inlet temperature 140 °C, outlet temperature 90 °C, blower speed 100%, peristaltic pump speed set at 40% (corresponding to a feed flow rate of approximately 600 L/h), with 6 automatic nozzle cleaning cycles performed.

An appropriate amount of microcapsules was placed into a 10 mL graduated cylinder and filled up to the 10 mL mark. The cylinder was then tapped/vibrated to achieve packing. After that, the microcapsules were poured out and weighed. The bulk density (ρb) of the microcapsules, expressed as the mass per 10 mL unit volume, was calculated according to Equation

(1) $$\rho_{b}\left(g/mL\right)=\left(M_{2}-M_{1}\right)/10$$

where **M_1_** is the mass of the empty cylinder, and **M_2_** is the mass of the cylinder together with the microcapsules.

### 2.8. Statistical Analysis

All data are presented as mean ± standard deviation. Differences among means were evaluated by one-way analysis of variance (ANOVA) followed by Duncan’s multiple range test using SPSS 27.0 and Origin 2019 software. All measurements were performed in triplicate.

## 3. Results and Discussion

### 3.1. Average Particle Size of Complexes

**Figure 2:**
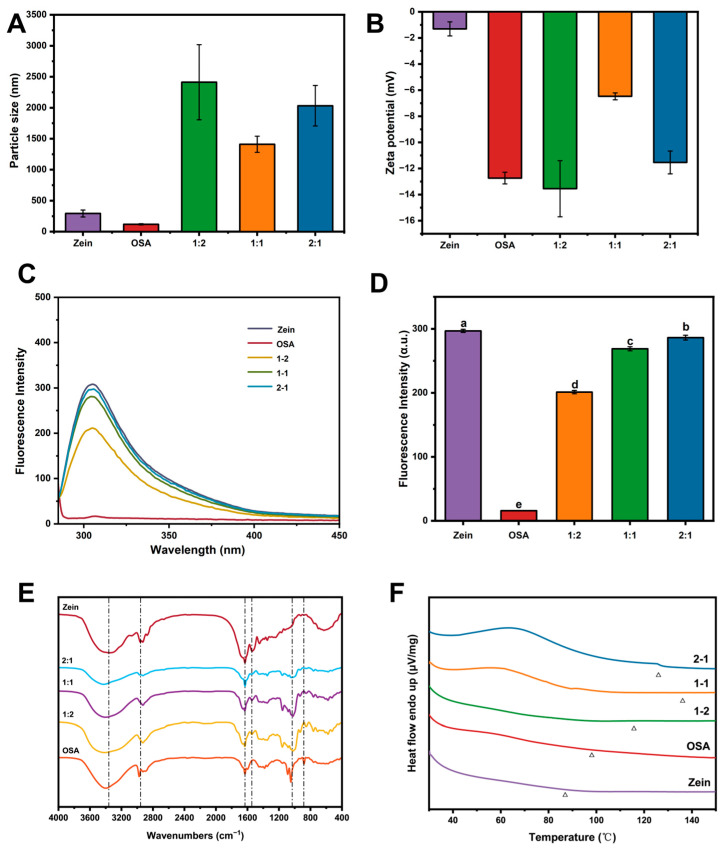
Characterization of Zein, OSA-starch, and complexes prepared at different mass ratios (Zein:OSA starch 2:1, 1:1, and 1:2). (**A**) Particle size; (**B**) Zeta potential; (**C**) Fluorescence intensity; (**D**) Fluorescence emission peak; (**E**) FTIR spectra; (**F**) DSC spectra. Within the same parameter to be used group, distinct lowercase letters (a–e) denote significant variations (*p* < 0.05) in the mean values.

Particle size serves as an indicator of the aggregation state and spatial conformation of Zein. Following complexation with OSA-starch, a significant increase in particle size is observed (Figure 2A), suggesting a modification in the associative behavior between the two components. In comparison to individual Zein or OSA-starch, the formed complexes generally possess larger dimensions [22] among these, the Zein-OSA starch (1:1) complex displayed the smallest average diameter (1410 ± 130.62 nm). As shown in Figure 2B, both Zein and OSA-starch exhibited negative surface charges, with OSA-starch carrying a greater magnitude of negative charge. Upon complex formation, the zeta potential of all composites shifted to more negative values with higher OSA-starch content [23]. Notably, the 1:1 complex attained a moderately negative zeta potential of approximately −6.47 ± 0.27 mV. This rearrangement is attributed to an electrostatically driven reorganization of interfacial charges, arising from the attraction between positively charged patches on Zein and the anionic moieties of OSA-starch. The resulting zeta potential values reside within a range that promotes colloidal stability through an electrostatic repulsion mechanism.

### 3.2. Fluorescence Spectroscopy

Interactions between proteins and other molecules often induce distinct changes in fluorescence emission spectra, including quenching effects and shifts in peak positions. These spectral variations can thus serve as a sensitive probe for detecting conformational changes in proteins driven by molecular associations [16]. As illustrated in Figure 2C,D, the Zein-OSA complex displayed a maximum emission wavelength at 309 nm, with a gradual decline in fluorescence intensity as the proportion of OSA starch increased relative to Zein. Further supported by Figure 2D, the fluorescence intensities of Zein and its complexes at different mass ratios differed significantly (*p* < 0.05), following the order: Zein > 2:1 > 1:1 > 1:2. Based on intrinsic fluorescence spectroscopy, Wani et al. [24] attributed such decreases in fluorescence emission to the binding of polysaccharides within hydrophobic regions of Zein. This interaction exposes buried chromophores, such as tryptophan and tyrosine, to a more polar environment, leading to fluorescence quenching—a phenomenon indicative of protein conformational reorganization through hydrophobic interactions and the subsequent formation of a stable composite. Similarly, in a thiolated chitosan-Zein-capsaicin system, variations in fluorescence intensity have been linked to chromophore microenvironmental changes mediated by intermolecular forces such as hydrogen–bonding and hydrophobic encapsulation [25], further underscoring the utility of fluorescence spectroscopy in characterizing protein-polysaccharide interactions.

### 3.3. FTIR Spectrogram Analysis of Complexes

Changes in protein functional groups can be detected by analyzing characteristic absorption peaks using Fourier transform infrared (FTIR) spectroscopy, a technique well-suited for studying interactions between proteins and polysaccharides. As shown in Figure 2E, the FTIR spectra of the three Zein-OSA complexes prepared at different ratios displayed no new characteristic peaks. However, alterations in the intensity and broadening of existing absorption bands were observed, indicating that although no new chemical compounds were formed upon complexation, molecular interactions between OSA and Zein did occur, leading to modifications in spectral features [26].

In the amide A region (3000–3600 cm^−1^), associated with hydrogen–bonding, the addition of OSA altered the peak shape and width, accompanied by a noticeable red shift near 3348.2 cm^−1^. These changes suggest the presence of hydrogen–bonding interactions between OSA and Zein [27]. Variations were also evident in amide I (~1631.4 cm^−1^) and amide II (~1537.1 cm^−1^) bands from Zein. With increasing OSA content, the amide I peak showed a blue shift, which may be attributed to hydrophobic interactions that contribute to alterations in secondary structure from Zein [26]. In contrast, changes in the amide II region were less distinct. Furthermore, the characteristic C=O stretching peak of OSA near 1614 cm^−1^ was absent in the composite spectra [20], further supporting molecular association between the two components. The absence of this distinct peak is attributed to the involvement of OSA’s carboxylate groups in strong electrostatic interactions and/or hydrogen–bonding with protonated amino groups (–NH_3_^+^) of Zein, particularly under the experimental pH conditions. Such interactions alter the local electron density and vibrational environment of the carbonyl group, leading to signal broadening, attenuation, or overlap with the adjacent amide I band of Zein (~1631 cm^−1^), rather than generating a new covalent bond. This phenomenon is commonly observed in associative complexes where charged biopolymers interact, and it further corroborates that OSA is not merely physically mixed but is intimately associated with Zein at the molecular level, contributing to the formation of a cohesive interfacial structure. Collectively, these spectral modifications—including shifts in amide bands and the disappearance of the OSA carbonyl signal—demonstrate the involvement of hydrogen–bonding and hydrophobic effects, thereby confirming the successful formation of OSA-Zein complexes as evidenced by FTIR analysis.

### 3.4. DSC Analysis of Complex

Based on the differential scanning calorimetry (DSC) analysis (Figure 2F), the thermal transition behavior of the complexes revealed a distinct enhancement in stability. The characteristic endothermic peak temperatures (Tp) were observed at approximately 85.0 °C for pure Zein (ascribed to protein denaturation). Similarly, Zein showed an endothermic peak at 88 °C as reported in Batool et al. [28] and 90.0 °C for OSA starch, which is associated with gelatinization and melting of crystalline regions as reported by Xie et al. [29]. In contrast, the 1-2 Zein-OSAstarch composite exhibited a significantly shifted Tp to about 115.0 °C. Notably, the 1–1 composite demonstrated the most pronounced improvement, with a major endothermic transition centered at ~145.0 °C. This marked increase of over 55 °C compared to the Zein indicates a substantial elevation in the thermal stability of the composite matrix. The sharp and well-defined peak suggests the formation of a more homogeneous and tightly integrated structure, likely due to optimized intermolecular interactions for hydrogen–bonding and hydrophobic forces between Zein and OSA starch [30]. The superior thermal resilience of the 1-1 complex implies a greater energy barrier required to disrupt its structure, making it a more robust candidate for applications involving thermal processing or storage. As the OSA starch content increased from the 2:1 to the 1:2 ratio, the peak temperature shifted systematically toward higher values, indicating that the overall hydrophilicity and water-binding strength of the composites are governed by the OSA starch proportion [31].

### 3.5. Moisture Content

**Figure 3:**
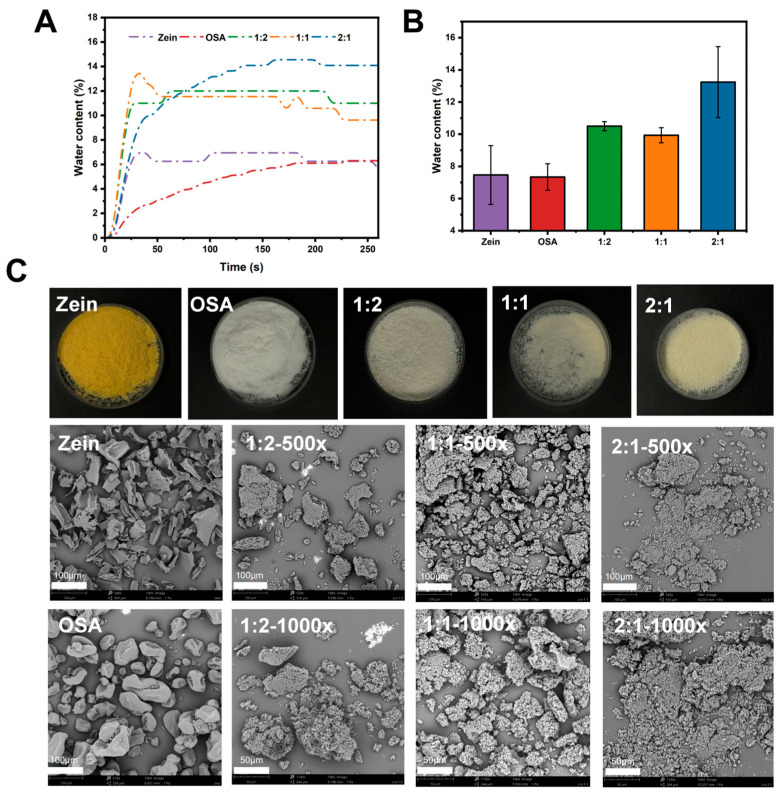
Water content and morphological characteristics of Zein-OSA starch complexes at different mass ratios (Zein:OSA 2:1, 1:1, 1:2). (**A**) Water content diagram; (**B**) Water content; (**C**) Sample appearance image and SEM image.

The moisture barrier performance was assessed of moisture content change, where the moisture content was determined gravimetrically by drying samples to constant weight at 105 °C. As shown in Figure 3A, the dynamic vapor sorption profiles of complexes prepared at different mass ratios revealed a gradual increase in moisture uptake until equilibrium was approached. Among all formulations, the complex with a 1:1 Zein-OSA ratio demonstrated the most gradual moisture loss kinetics and ultimately reached the lowest equilibrium moisture content. The correlation between equilibrium moisture content and composite composition is further quantified in Figure 3B. A pronounced minimum (9.94 ± 0.47%) was observed at the 1:1 mass ratio, indicating a non-monotonic dependence of hydration behavior on the blending proportion. This trend suggests that both hydrophobicity and structural compactness of the complexes are critically influenced by the component ratio, with the 1:1 formulation exhibiting the lowest affinity for atmospheric moisture. Collectively, these findings confirm that the Zein-OSA complex prepared at a 1:1 mass ratio possesses the most effective intrinsic moisture-barrier characteristics, also according to the report by Wu et al. [32], a higher moisture content in starch enhances shear resistance, which influences the emulsion formation process. Meanwhile, the partially gelatinized starch-protein composite shell functions as a water penetration barrier, exhibiting improved heat resistance and hydrophobicity properties that further facilitate the stabilization of the emulsion.

### 3.6. SEM

The particle morphologies of Zein and OSA starch, before and after complex treatment (SEM, Figure 3C). SEM images at 500× magnification revealed that untreated Zein and SA-starch exhibited a flaky structure and an aggregated granular structure, respectively. During the preparation of the composite, the input of mechanical energy significantly altered the morphology of the resulting composite particles. During processing, the original coarse granular structures were largely disrupted and transformed into a greater quantity of smaller composite grains [33]. The granular structure became looser and more porous, with the particle morphology turning irregular with complexes formed. This transformation is attributed to the mechanical forces from the strong forces involved in the formation of complexes, which introduced a higher density of structural defects into the starch granules. These defects promoted fracture, breaking the granules into smaller particles and fragments, thereby reducing the overall particle size and significantly increasing the specific surface area of the powder. The resulting damaged particles tended to agglomerate into clusters or adhere to the surface of larger granules, as reported by Luo et al. [34].

At a mass ratio of 1:1, the complex exhibited a loosely organized lamellar structure. In contrast, at higher OSA-starch proportions, the lamellar morphology appeared more fragmented and compact. Furthermore, the amount of OSA-starch added influenced the phase-separation behavior of the system. At lower OSA levels, phase separation was limited, yielding relatively homogeneous and irregular flake-like structures. Conversely, higher OSA contents promoted more pronounced phase separation, leading to altered interfacial properties and phase composition, ultimately resulting in well-defined, continuous lamellar architectures [35].

### 3.7. Microstructure Observations of Emulsion

**Figure 4:**
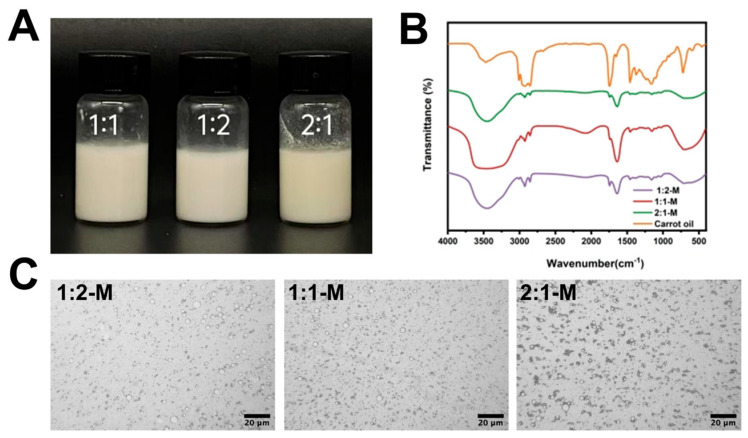
Preparation and characterization of carrot oil emulsions stabilized by Zein-OSA starch complexes (Zein:OSA 2:1, 1:1, 1:2). (**A**) Emulsion appearance diagram; (**B**) FTIR of Carrot oil and Emulsion; (**C**) microscope image of emulsion.

Prior to microscopic evaluation, the macroscopic stability of the emulsions was first examined. Visual inspection confirmed that all freshly prepared emulsions displayed no visible phase separation or creaming (Figure 4A), demonstrating good initial colloidal stability. The emulsions presented a homogeneous, milky-white appearance, consistent with efficient light scattering from uniformly dispersed oil droplets maintained at sufficient inter-droplet distances to inhibit flocculation and coalescence, key factors supporting long-term stability. Microscopic analysis further corroborated this observation, revealing a well-distributed oil droplet population without signs of aggregation (Figure 4C). The observed morphology suggests that the stabilizing complexes likely assemble into a cohesive interfacial network, effectively encapsulating the oil droplets and preventing their coalescence, thereby underpinning the high stability noted both macroscopically and microscopically.

### 3.8. FTIR

The chemical structures of carrot oil and Zein-OSA starch composite emulsions were analyzed by Fourier-transform infrared (FT-IR) spectroscopy, as shown in Figure 4B. The spectrum of pure carrot oil (Figure 4B) exhibited characteristic absorption bands around 2920 cm^−1^ and 2850 cm^−1^ (asymmetric and symmetric C–H stretching of alkyl chains), 1745 cm^−1^ (C=O stretching of triglycerides), and 1160 cm^−1^ (C–O stretching of ester groups) [36]. The corresponding spectra of the composite emulsions (2:1-M, 1:1-M, and 1:2-M) are displayed in the right panel. Notably, the characteristic lipid bands associated with C–H and C=O stretching were markedly attenuated in all composite spectra compared to those of pure oil. This attenuation reflects the effective encapsulation of carrot oil within the Zein-OSA matrix, which masks the infrared signals of the core material. In summary, FT-IR analysis confirmed the successful encapsulation of carrot oil and provided direct spectroscopic evidence of molecular interactions, primarily hydrogen–bonding and hydrophobic effects between Zein and OSA starch in the emulsion systems.

### 3.9. Stability of Emulsion

#### 3.9.1. Particle Size in Storge Time

**Figure 5:**
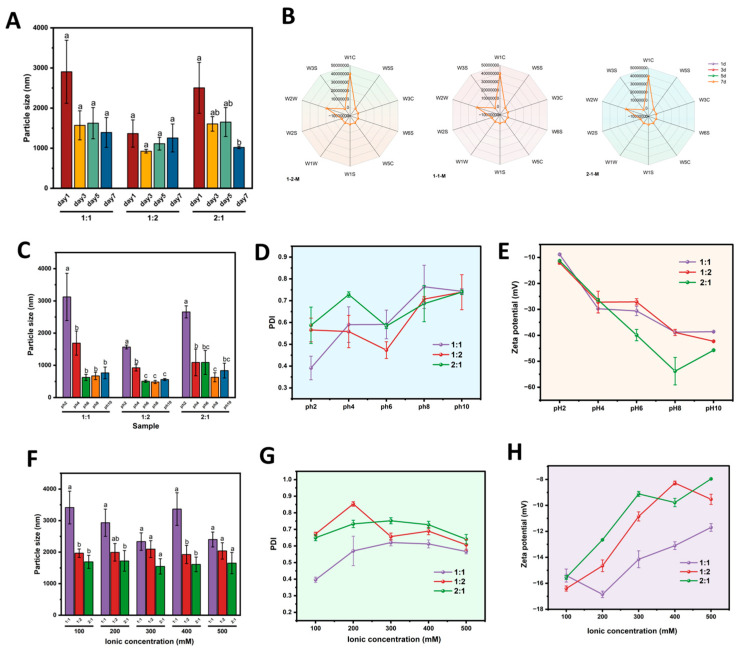
Stability of carrot oil emulsions stabilized by Zein-OSA starch complexes (mass ratios: 2:1, 1:1, 1:2) under different conditions. (**A**) Average droplet size (storage time); (**B**) emulsion electronic nose response image; (**C**) particle size under different pH conditions; (**D**) PDI under different pH conditions; (**E**) potential under different pH conditions. (**F**) particle size under different ion concentrations; (**G**) PDI under different ion concentrations; (**H**) potential under different ion concentrations. Within the same parameter to be used group, distinct lowercase letters (a–c) denote significant variations (*p* < 0.05) in the mean values.

Figure 5A shows the variation in droplet size of the emulsions during storage. Emulsions stabilized with the 1:1-M and 1:2-M composite ratios showed no significant change in particle size over time (*p* > 0.05). In contrast, the emulsion prepared with the 2:1-M composite exhibited a significant decrease in droplet size from day 1 (2504.01 ± 634.55 nm) to day 7 (1020.79 ± 31.09 nm) (*p* < 0.05). The instability of the internal droplets may contribute to the observed trend of decreasing droplet size associated with the increasing proportion, as reported by Pei et al. [37]. Throughout the storage period, the 1:1-M and 1:2-M emulsions maintained the smallest average particle size among all formulations, indicating their superior stability. According to the report by Zhao et al. [38], the improvements could be ascribed to the generation of a denser and antioxidant interfacial film as well as an interconnected network structure within the system.

#### 3.9.2. Electronic Nose

The volatile profiles of the emulsions during storage were analyzed daily using a 10-sensor electronic nose, with the response intensities presented in Figure 5B. All emulsions showed relatively high signals for sensors W1C (aromatics), W3S (alkanes and aliphatics), W2W and W2S (organic sulfides), W5C (ethanol and certain aromatics), and W3C (ammonia and aromatic compounds) [39]. When stored at 25 °C, significant changes in volatile profiles were observed between days 3 and 7 compared to day 1, characterized by elevated responses from sensors W1C and W2W during this period. In contrast, the three emulsion groups exhibited no significant changes in their volatile profiles under electronic nose detection, indicating that the emulsion preparation effectively improved the control over volatile release.

#### 3.9.3. Analysis of Aroma Components by GC-MS

The primary aroma compounds in carrot oil emulsions stabilized by three different protein–polysaccharide composite ratios, as identified by GC-MS, are summarized in Table S1. The retention capacity for these aroma components varied significantly depending on the composite formulation. The dominant aroma compounds detected were carotol, daucol, and β-bisabolene, which align with previous reports [40]. As shown in Table S1, the content of carotol—a key aroma compound—exhibited an initial increase followed by a gradual decline in both the 1:1-M and 1:2-M emulsions, with relatively small overall variation. In contrast, the 2:1-M emulsion displayed a sharp decrease in carotol content during later storage. Daucol levels remained moderately stable in the 1:1-M emulsion, showed minimal fluctuation in the 1:2-M emulsion, but varied considerably with poor stability in the 2:1-M emulsion in later stages. β-Bisabolene remained relatively stable throughout storage in both the 1:1-M and 1:2-M emulsions. Regarding secondary components, furfuryl alcohol was present at low concentrations across all emulsions, with a more pronounced decrease observed in the 2:1-M emulsion during extended storage. Methyl cis-6-octadecenoate and trans-2,4-decadienal were consistently detected at trace levels in all formulations and showed little change over time. Linoleic acid content was initially low in the 1:1-M and 1:2-M emulsions but increased gradually during storage, whereas in the 2:1-M emulsion, it rose sharply in the early stage before declining in the later period.

Overall, the 1:1-M formulation exhibited the highest stability for key aroma components such as carotol and daucol, followed by the 1:2-M formulation. In contrast, the 2:1-M formulation tended to promote the early release of linoleic acid and displayed lower stability for several components during extended storage. These variations may be attributed to the influence of the OSA-Zein ratio on the structural integrity of the emulsion, which in turn modulates the retention, release, or degradation kinetics of the volatile compounds.

#### 3.9.4. pH Stabiliy

The pH stability of emulsions stabilized by Zein-OSA starch complexes at different mass ratios was evaluated over a pH range of 2 to 10 (Figure 5C–E). The mean droplet diameter for all composites reached its maximum at pH 2 (*p* < 0.05), decreased from pH 2 to 6, and then increased again up to pH 10. The emulsion with a 1:2-M ratio exhibited the smallest average droplet size (0.5 μm) at pH 6. According to PDI analysis, the most uniform droplet distribution for the 1:1 complex is observed at pH 2 (PDI = 0.392), whereas for the 1:2 and 2:1 complexes, uniformity is highest at pH 6 (PDI = 0.473 and 0.583, respectively). Zeta potential measurements indicated moderate to good electrostatic stability (absolute values between −25 and −50 mV) across pH 4 to 10 for all emulsions. Integrating droplet size, PDI, and zeta potential data identified the emulsion prepared with a 1:1 Zein:OSA starch ratio at pH 6 as the most stable formulation.

**Figure 6:**
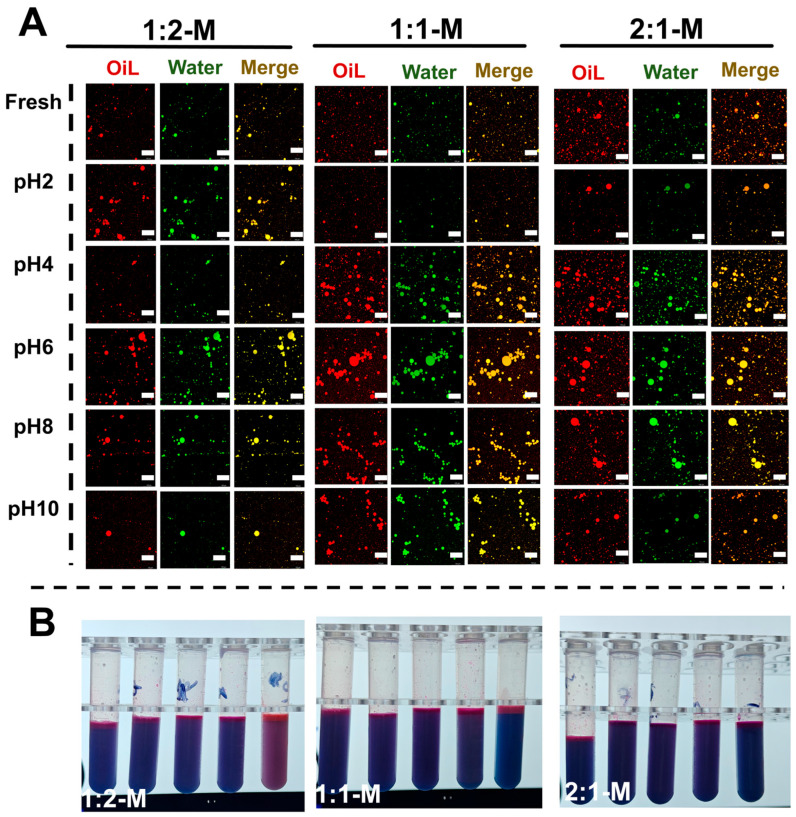
Stability of carrot oil emulsions stabilized by Zein-OSA starch complexes (mass ratios: 2:1, 1:1, 1:2) under different pH conditions. (**A**) CLSM images; (**B**) macroscopic appearance.

The microstructural and physical stability of Zein-OSA starch-stabilized emulsions (1:2-M, 1:1-M, 2:1-M) across pH 2–10 were further analyzed using confocal laser scanning microscopy (CLSM), with corresponding visual observations shown in Figure 6A,B. CLSM images revealed distinct pH-dependent structural changes, consistent with previous reports [41]. Under extreme pH conditions (pH 2 and 10), all emulsions exhibited droplet coalescence, flocculation, or phase separation, correlating with macroscopic instability (Figure 6B). Within the pH 4–8 range, the emulsion microstructure improved significantly. While the 1:1-M complex maintained relatively stable structures across a broader pH range, the 2:1-M complex showed notable instability under acidic conditions (pH 2–4).

#### 3.9.5. NaCl Stability

The stability of emulsions stabilized by Zein-OSA starch complexes was evaluated under varying ionic strengths (0–0.5 M NaCl), as shown in Figure 5F–H. While droplet size did not exhibit a clear trend with increasing NaCl concentration for any composite ratio, significant differences were observed among the different Zein:OSA starch formulations. Across all ionic strengths, the emulsion stabilized by the 1:1 complex consistently showed the largest droplet size. The polydispersity index (PDI) was lowest for the 1:1-M emulsion at 0.1 M NaCl (PDI = 0.397), indicating the most uniform droplet distribution under this condition. Zeta potential analysis revealed a general decline in absolute values with increasing ionic strength for all emulsions, consistent with electrical double-layer compression [20]. The highest zeta potentials were recorded at low to moderate ionic strengths (0.1–0.2 M), with the 1:1-M emulsion reaching a peak value of −16.85 mV at 0.2 M. These results suggest that, within the tested range, the composite ratio exerts a greater influence on emulsion properties than ionic strength, and that the 1:1 complex provides the most effective electrostatic stabilization [42].

**Figure 7:**
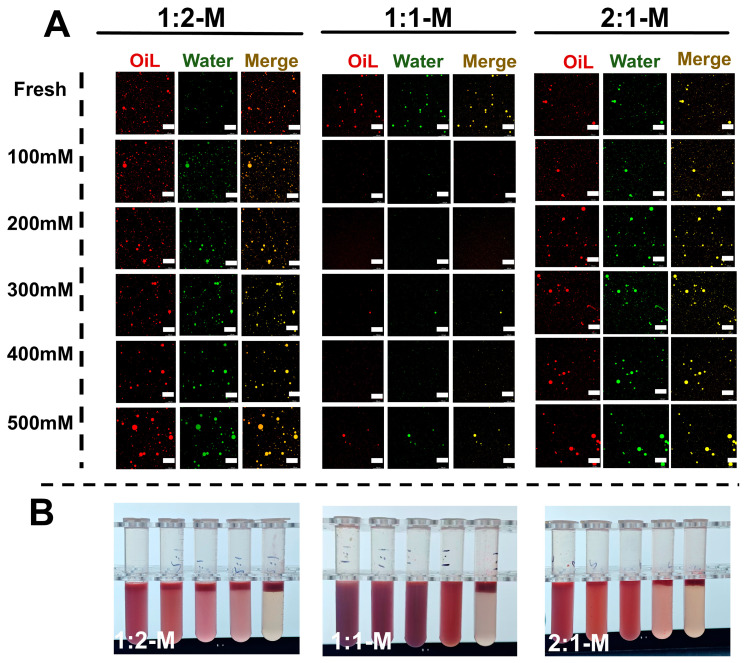
Ionic strength stability of Zein-OSA starch complex-stabilized carrot oil emulsions (mass ratios: 2:1, 1:1, 1:2). (**A**) CLSM images; (**B**) macroscopic photos.

CLSM observations further revealed distinct microstructural responses to ionic strength. The 1:2-M emulsion exhibited relatively uniform droplet distribution at lower salt concentrations (≤0.2 M), with only slight aggregation observed. However, as salt concentration increased to 0.3–0.5 M NaCl, noticeable droplet coalescence and structural heterogeneity developed, indicating reduced stability under high ionic strength. In contrast, the 2:1-M emulsion underwent pronounced droplet coalescence and phase separation at NaCl concentrations ≥0.4 M. The 1:1-M emulsion exhibited intermediate stability, largely retaining its microstructure up to 0.3 M NaCl. Macroscopic appearance correlated well with CLSM images: only the 1:2-M emulsion remained visually stable at all ionic strengths, whereas the 2:1-M formulation showed evident creaming and phase separation above 0.2 M NaCl, as shown in Figure 7. These findings underscore the superior salt tolerance of the OSA-rich composite, which can be attributed to enhanced steric and electrostatic stabilization, confirming its robustness as an emulsion stabilizer under varying ionic conditions.

### 3.10. Rheological Properties of Emulsions in Different Storage Conditions

**Figure 8:**
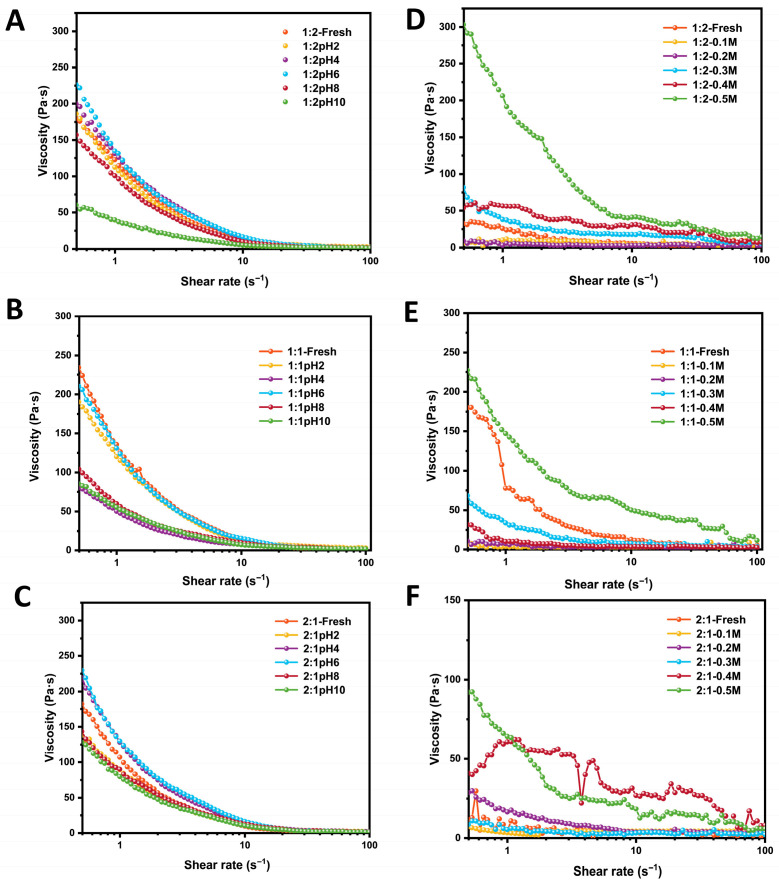
Rheological properties of emulsions stabilized by Zein-OSA starch complexes at different mass ratios (2:1, 1:1, 1:2) under varying pH (**A**–**C**) and ionic strength conditions (**D**–**F**).

Apparent viscosity is a critical parameter affecting emulsion stability, as higher viscosity generally correlates with enhanced stability. This study systematically evaluated the viscosity behavior and stability of emulsions under varying pH (2–10) and ionic strength (0–0.5 M) conditions using rheological approaches. The viscosity profiles of emulsions stabilized by different composite particles are presented in Figure 8A–C at different pH levels. Under all tested conditions, the apparent viscosity exhibited a sharp decline as the shear rate increased from 0.1 to 10 s^−1^, followed by a more gradual decrease between 10 and 100 s^−1^. As reported by Wang et al. [43], under extreme pH conditions, the ionization state of emulsifiers is altered, leading to aggregation and desorption near the isoelectric point, which compromises the integrity of the interfacial film. This shear-thinning behavior is characteristic of non-Newtonian fluids, indicating that the Zein-OSA starch composite-stabilized emulsions possess non-Newtonian rheological properties. Shear-thinning can reduce energy consumption during processing while maintaining structural integrity, thereby contributing to emulsion stability [17], a finding consistent with the results of Weng et al. [44]. Within the low shear-rate range (0.1–10 s^−1^), the apparent viscosity of emulsions stabilized by all composite ratios reached its maximum at pH 6. Comparison of peak apparent viscosities across Figure 8A–C indicates that the emulsion with a 1:1 composite ratio achieved the highest viscosity. Moreover, apparent viscosity generally increased with a higher proportion of OSA starch in the composite emulsifier, which aligns with the conclusions of [45]. The enhanced viscosity observed for the 1:1 composite may be attributed to the formation of a more cohesive network structure at this ratio, which improves interfacial stability, reinforces resistance to shear, and consequently elevates viscosity.

A similar trend was observed under varying ionic strength conditions (Figure 8D–F). In high ionic strength environments, compression of the electrical double layer around droplets weakens electrostatic repulsion, thereby inducing flocculation and coalescence. Increasing ionic strength systematically reduced viscosity across all composite ratios, which can be explained by electrical double-layer compression that diminishes electrostatic repulsion between particles and weakens the network structure. However, at higher ionic strengths (>0.2 M), pronounced double-layer compression leads to dominance of van der Waals attraction, resulting in irreversible coalescence, droplet growth, and a marked decrease in viscosity, accompanied by a shift toward Newtonian flow behavior. Notably, the 1:1 ratio emulsion achieves an optimal structural balance. Its moderate interfacial area and film thickness allow the formation of a sufficiently compact adsorbed layer to resist emulsifier inactivation caused by pH changes, while providing adequate steric hindrance and residual electrostatic repulsion to mitigate flocculation pressure from increased ionic strength. Therefore, it demonstrates the most balanced and robust rheological behavior and storage stability across a wide range of environmental conditions.

### 3.11. Characterization of Microcapsules

Microcapsules serve as effective delivery systems for lipophilic active compounds. Encapsulation via spray drying converts liquid emulsions into free-flowing powders, significantly enhancing their handling, storage stability, and applicability in food products.

**Figure 9:**
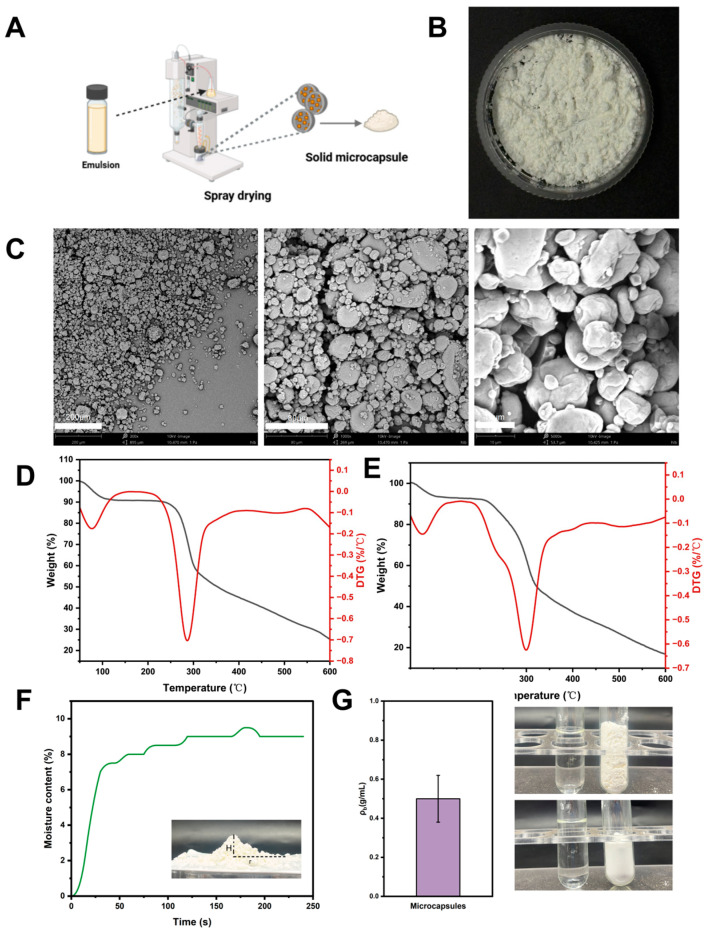
Preparation and characterization of spray-dried microcapsules derived from carrot oil emulsions stabilized by Zein-OSA starch complexes at different mass ratios (2:1, 1:1, 1:2). (**A**) microcapsule preparation image; (**B**) microcapsule appearance image; (**C**) scanning electron microscope image; (**D**) complex TGA curve; (**E**) microcapsule TGA image; (**F**) microcapsule water activity image; (**G**) microcapsule packing density image.

Following spray-drying of the emulsion (Figure 9A), a free-flowing white powder is obtained (Figure 9B). SEM analysis (Figure 9C) showed that the resulting microcapsules possessed a wrinkled surface morphology. This characteristic texture is a direct result of the rapid dehydration and shrinkage of the emulsion droplets during the spray-drying process. Notably, the primary wall material, Zein-OSA starch granules (inset in Figure 3C), themselves exhibited a rough and partially cracked surface, which may have influenced the interfacial properties and contributed to the final capsule morphology upon drying [46]. Importantly, despite sharing some morphological irregularities with the Zein-OSA starch matrix, the microcapsule surfaces showed no evident fractures or cracks. This structural preservation suggests that the carrot oil core was effectively encapsulated and protected during drying. The observed surface wrinkles can be mechanistically explained by the rapid evaporation of water from the emulsion droplets during spray drying. Similar reports were also presented by Wang et al. [47], to illustrate that the pepper oil, after being prepared into microcapsules, has a better release effect.

Same reported by Wang et al. [21] as the droplet surface contracts quickly under high-temperature conditions, the forming solid shell undergoes non-uniform shrinkage, leading to the characteristic wrinkled topography without compromising encapsulation integrity. These morphological outcomes highlight the role of both chemical modification (surface roughening of OSA starch) and process-induced physical changes (wrinkling during spray drying) in determining the final microstructure of the encapsulates, while the absence of cracks corroborates successful oil retention and core protection.

The thermal behavior of selected emulsions and the resulting microcapsules was evaluated by thermogravimetric analysis (TGA, Figure 9D,E). The thermal degradation temperature, determined from the peak in the derivative thermogravimetric (DTG) curve, reflects the thermal stability of the samples, with higher values indicating greater stability. The microcapsules exhibited a significantly higher degradation temperature (299.79 °C) compared to the emulsion (286.75 °C). Additionally, the microcapsule system showed a moisture content of 9.0 ± 0.5% and a packing density of 0.49 ± 0.01 g/mL, indicating a loose, porous structure with considerable inter-particle voids.

### 3.12. Molecular Docking

**Figure 10:**
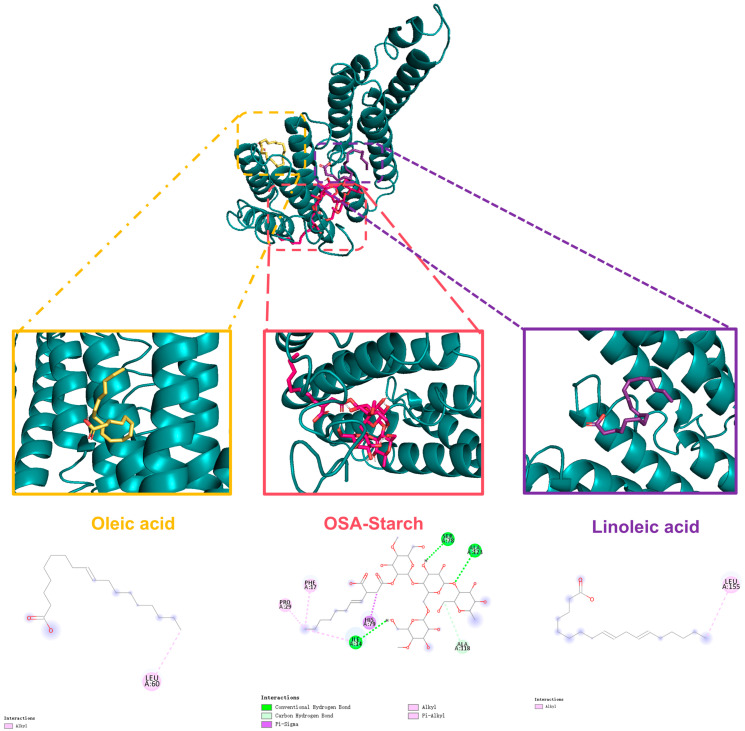
Visualization of the binding interactions between oleic acid/linoleic acid and the Zein-OSA starch (1:1) complex through molecular docking.

Molecular docking was employed to investigate the binding interactions between fatty acids and the Zein-OSA complex (Figure 10). The stability of these interactions was evaluated using binding energy, where lower (more negative) values indicate stronger binding affinity, consistent with established computational approaches [48]. The docking simulation was conducted using the major fatty acids present in carrot oil as ligands. Initially, the Zein-OSA complex itself was optimized, exhibiting a stable conformation with a binding energy of −6.4 kcal/mol. Subsequent docking with linoleic acid (LA) and oleic acid (OA)-the predominant fatty acids in carrot oil-yielded optimal conformations with binding energies of −3.5 kcal/mol and −4.2 kcal/mol, respectively.

These results suggest that both LA and OA can bind spontaneously to the Zein-OSA complex, with OA showing a moderately stronger affinity. The observed binding energies are consistent with the formation of stable complexes, potentially involving hydrogen–bonding, hydrophobic interactions, or van der Waals forces between the fatty acids and the composite matrix. This provides a molecular-level insight into how the Zein-OSA system may encapsulate and retain lipophilic bioactive components such as carrot oil fatty acids.

## 4. Conclusions

This study establishes a novel and rational approach for encapsulating sensitive carrot seed oil using food-grade, binary complexes of Zein and OSA-modified starch, which systematic demonstrated that the Zein-to-OSA starch mass ratio serves as a precise, tunable parameter to dictate the hierarchical structure and functionality of the delivery system across molecular, colloidal, and application scales, a relationship not previously elucidated for this specific oil and composite matrix. At the molecular level, the 1:1 (*w*/*w*) Zein-OSA starch complex was identified as the optimal encapsulating matrix. This composition exhibited the strongest intermolecular interactions via hydrogen–bonding and hydrophobic forces, resulting in a hybrid complex with superior thermal stability and effective moisture barrier properties, which are critical for protecting the oil from degradation. Meanwhile, at the colloidal interface, the 1:1 (*w*/*w*) complex emerged as the most effective Pickering stabilizer. As a Pickering emulsifier, the 1:1 complex conferred superior colloidal stability, producing emulsions with fine droplet size, notable salt tolerance, and enhanced aroma retention. All emulsions exhibited desirable shear-thinning rheology and adjustable stability across a range of pH conditions, confirming their robustness. Finally, leveraging these fundamental insights, we successfully translated the optimal liquid Pickering emulsion (stabilized by the 1:1 complex) into solid, free-flowing microcapsules via spray-drying. This critical advancement overcomes the inherent limitations of liquid emulsions, providing a physically stable powder with markedly improved thermal stability for the encapsulated oil, thereby dramatically broadening its practical application potential.

In summary, this work provides a comprehensive proof-of-concept that the composition of Zein-OSA starch complexes can be rationally modulated to design carriers tailored for specific functional endpoints, be it maximizing molecular encapsulation, optimizing colloidal stability, or enabling solid powder formation. We have thus developed a sustainable, food-grade platform technology that effectively addresses the key challenges of stability, volatility, and dispersibility associated with carrot seed oil and, by extension, other sensitive lipophilic bioactives. While this study demonstrates the potential of the delivery system for enhancing the stability and bioaccessibility of the bioactive compound, several limitations should be noted. Future work should focus on applications in complex food products, and the development of co-delivery systems for synergistic actives.

## Data Availability

The data presented in this study are available on request from the corresponding author.

## Supplementary Materials

The following supporting information can be downloaded at: https://www.mdpi.com/article/10.3390/foods15030435/s1, Table S1: The trend of changes in the main volatile components during storage in different emulsion systems.
