# Supplementary material for: Fabricating Zein-OSA Starch Complexes as Multifunctional Carriers for Carrot Oil
Source: Foods. 2026 Jan 24;15(3):435. doi: 10.3390/foods15030435 (PMC12897285; doi:10.3390/foods15030435)
Supplement: Supplementary file 1 [file foods-15-00435-s001.zip › foods-4087732-supplementary.pdf]

Table S1. The trend of changes in the main volatile components during storage in different emulsion systems

NO	Composition	Time (min)	1-2-M				1-1-M				2-1-M			
			1 Day	3 Day	5 Day	7 Day	1 Day	3 Day	5 Day	7 Day	1 Day	3 Day	5 Day	7 Day
1	2-Furanmethanol	43.425	0.83 ± 0.11^{Ae}	1.33 ± 0.09^{Ae}	0.72 ± 0.02^{BCf}	0.63 ± 0.04^{Cd}	0.82 ± 0.01^{Ae}	0.75 ± 0.04^{Be}	0.75 ± 0.03^{Bf}	0.64 ± 0.04^{Cd}	0.77 ± 0.02^{Ae}	0.68 ± 0.01^{Be}	0.66 ± 0.01^{Be}	0.24 ± 0.00^{Cc}
2	β-Bisabolene	48.595	8.20 ± 1.02^{Ac}	9.10 ± 0.12^{Ac}	8.24 ± 0.44^{Ac}	6.44 ± 0.33^{Bc}	7.54 ± 0.14^{Bc}	5.11 ± 0.11^{Cd}	8.43 ± 0.43^{Ac}	7.99 ± 0.55^{ABc}	8.87 ± 0.11^{Ac}	4.35 ± 0.87^{Cd}	8.06 ± 0.59^{Ac}	2.06 ± 0.12^{Cc}
3	(E,E)-2,4-Decadienal	53.676	0.54 ± 0.01^{Ae}	0.56 ± 0.02^{Ae}	0.58 ± 0.01^{Af}	0.58 ± 0.01^{Ad}	0.50 ± 0.00^{De}	0.59 ± 0.01^{Ae}	0.56 ± 0.00^{Bf}	0.53 ± 0.01^{Cd}	0.28 ± 0.01^{Ce}	0.33 ± 0.01^{Be}	0.55 ± 0.03^{Ae}	0.10 ± 0.00^{Dc}
4	Carotol	62.772	19.73 ± 2.01^{Ab}	22.51 ± 2.11^{Ab}	21.01 ± 1.22^{Ab}	20.05 ± 2.44^{Ab}	17.48 ± 0.94^{Ab}	20.38 ± 0.83^{Ab}	20.61 ± 1.01^{Ab}	19.84 ± 3.87^{Ab}	20.28 ± 0.26^{Ab}	19.47 ± 1.45^{Ab}	20.21 ± 1.32^{Ab}	7.64 ± 0.34^{Bb}
5	Daucol	67.065	5.54 ± 0.09^{Bd}	5.92 ± 0.32^{Ad}	5.32 ± 0.02^{Be}	5.25 ± 0.05^{Bc}	4.73 ± 0.03^{Ad}	5.44 ± 0.32^{Ad}	5.41 ± 0.09^{Ad}	4.97 ± 0.88^{Acd}	5.37 ± 0.13^{Ad}	5.13 ± 0.11^{Ad}	5.06 ± 0.44^{Ad}	2.00 ± 0.02^{Bc}
6	Linoleic acid	68.183	0 ± 0.00^{De}	5.04 ± 0.22^{Cd}	6.24 ± 0.09^{Bd}	7.31 ± 0.11^{Ac}	0 ± 0.00^{Ce}	7.48 ± 0.23^{Ac}	2.17 ± 0.32^{Be}	2.31 ± 0.01^{Bcd}	0.27 ± 0.00^{Ce}	9.20 ± 0.62^{Ac}	9.49 ± 0.83^{Ac}	1.30 ± 0.01^{Bc}
7	(Z)- methyl ester-6-Octadecenoic acid	69.438	1.11 ± 0.02^{Ae}	0.87 ± 0.02^{Ce}	0.89 ± 0.01^{Cf}	1.04 ± 0.02^{Bd}	0.81 ± 0.01^{Ce}	0.89 ± 0.00^{BCe}	1.21 ± 0.11^{Aef}	0.95 ± 0.01^{Bd}	0.86 ± 0.01^{Be}	1.16 ± 0.12^{Ae}	0.81 ± 0.01^{Be}	0.35 ± 0.02^{Ce}
8	Others		64.05 ± 2.54^{Aa}	54.67 ± 4.55^{Ba}	57.00 ± 0.02^{Ba}	58.70 ± 3.76^{ABa}	68.12 ± 1.43^{Aa}	59.36 ± 2.59^{Ba}	60.86 ± 1.56^{Ba}	62.77 ± 5.76^{ABa}	63.30 ± 1.34^{Ba}	59.68 ± 1.83^{BCa}	55.16 ± 3.47^{Ca}	86.31 ± 4.28^{Aa}
